# Consensus-based recommendations for investigating clinical heterogeneity in systematic reviews

**DOI:** 10.1186/1471-2288-13-106

**Published:** 2013-08-30

**Authors:** Joel J Gagnier, Hal Morgenstern, Doug G Altman, Jesse Berlin, Stephanie Chang, Peter McCulloch, Xin Sun, David Moher

**Affiliations:** 1 Department of Orthopaedic Surgery, University of Michigan, MedSport, 24 Frank Lloyd Wright Drive, Ann Arbor, MI 48106, USA; 2 Department of Epidemiology, School of Public Health, University of Michigan, Ann Arbor, MI, USA; 3 Center for Statistics in Medicine, University of Oxford, Oxford, UK; 4 Research and Development, Johnson and Johnson Pharmaceutical, Philidelphia, PA, USA; 5 Agency for Healthcare Research and Quality, Rockville, MD, USA; 6 Centre for Evidence Based Medicine, University of Oxford, Oxford, UK; 7 Kaiser Permanente Center for Health Research and Oregon Evidence-based Practice Center, Oregon Health Sciences University, Portland, OR, USA; 8 Clinical Epidemiology Program, Ottawa Hospital Research Institute, Ottawa, ON, Canada; 9 Department of Epidemiology, University of Ottawa, Ottawa, ON, Canada

## Abstract

**Background:**

Critics of systematic reviews have argued that these studies often fail to inform clinical decision making because their results are far too general, that the data are sparse, such that findings cannot be applied to individual patients or for other decision making. While there is some consensus on methods for investigating statistical and methodological heterogeneity, little attention has been paid to clinical aspects of heterogeneity. Clinical heterogeneity, true effect heterogeneity, can be defined as variability among studies in the participants, the types or timing of outcome measurements, and the intervention characteristics. The objective of this project was to develop recommendations for investigating clinical heterogeneity in systematic reviews.

**Methods:**

We used a modified Delphi technique with three phases: (1) pre-meeting item generation; (2) face-to-face consensus meeting in the form of a modified Delphi process; and (3) post-meeting feedback. We identified and invited potential participants with expertise in systematic review methodology, systematic review reporting, or statistical aspects of meta-analyses, or those who published papers on clinical heterogeneity.

**Results:**

Between April and June of 2011, we conducted phone calls with participants. In June 2011 we held the face-to-face focus group meeting in Ann Arbor, Michigan. First, we agreed upon a definition of clinical heterogeneity: Variations in the treatment effect that are due to differences in clinically related characteristics. Next, we discussed and generated recommendations in the following 12 categories related to investigating clinical heterogeneity: the systematic review team, planning investigations, rationale for choice of variables, types of clinical variables, the role of statistical heterogeneity, the use of plotting and visual aids, dealing with outlier studies, the number of investigations or variables, the role of the best evidence synthesis, types of statistical methods, the interpretation of findings, and reporting.

**Conclusions:**

Clinical heterogeneity is common in systematic reviews. Our recommendations can help guide systematic reviewers in conducting valid and reliable investigations of clinical heterogeneity. Findings of these investigations may allow for increased applicability of findings of systematic reviews to the management of individual patients.

## Background

There are several possible sources of variability or heterogeneity among studies that are included in systematic reviews and meta-analyses. Variability in the participants, the types or timing of outcome measurements, and intervention characteristics has been termed clinical heterogeneity [1]; variability in the trial design or execution is commonly termed methodological heterogeneity [2]; variability in summary treatment effect estimates among trials is termed statistical heterogeneity [1]. These sources of heterogeneity are not necessarily mutually exclusive. For example, statistical heterogeneity may arise from clinical or methodological heterogeneity, from other unknown or unrecorded trial characteristics, or it may be due to chance.

Another way of characterizing sources of heterogeneity of the estimated effect (association) among studies in meta-analyses is outline in the following 3 items. 1. True effect variation among studies (“clinical heterogeneity”) – due to; a. individual characteristics in the population (e.g., age, disease severity, comorbidities) – i.e., individual modifiers of the treatment/exposure effect; b. population context (e.g., type of setting or organization, practice pattern) – i.e., contextual modifiers of the treatment/exposure effect; c. type or nature of the treatment or exposure (e.g., dose or frequency); d. choice of outcome measure; e. choice of effect measure and follow-up period (e.g., risk or rate ratio, risk or rate difference). 2. Within-study estimation error variation among studies (“methodological heterogeneity”) – due to; a. Random error – low power for detecting effects due to small sample sizes, rare outcome events, or weak associations; and poor estimation precision (wide confidence intervals); b. Bias due to confounding (e.g., confounding by indication), selection or participation of subjects (which depends on the outcome or outcome risk), or measurement error; c. Bias due to model misspecification (e.g., due to ignored interactions or sparse data); d. Temporal ambiguity between hypothesized predictors and outcomes (e.g., due to design limitations, statistical limitations, or reverse causation). 3. Between-study estimation error in the meta-analysis (“methodological heterogeneity”)– due to: a. Aggregated data from studies used as covariates in meta-regression or subgroup analysis (e.g., mean age or proportion of males) – a form of ecologic bias; b. Misspecification of the meta-regression model; c. Random error – low power and precision due to a small number of studies; d. Publication bias – selective reporting and publication of results.

In general, clinical heterogeneity may arise from differences in participant characteristics (i.e., Patient-level variables; e.g., sex, age, baseline disease severity, ethnicity, comorbidities), types or timing of outcome measurements, and intervention characteristics (i.e., Study level variables; e.g., dose and frequency of dose) [1]. Clinical heterogeneity can cause substantively important statistical heterogeneity, varying summary effect estimates and associated conclusions, potentially misleading decision makers and other end-users of systematic reviews.

Systematic reviews and meta-analyses are frequently recognized as the best available evidence for decisions about health-care management and policy [3,4]. As such, results of systematic reviews are incorporated into clinical practice guidelines [5], sometimes required by granting agencies in funding applications [6] and a growing body of experts devote considerable time to completing them [7]. In spite of the documented importance of systematic reviews, it appears health-care professionals and policy makers infrequently use systematic reviews to guide decision-making [8]. This may be due to several factors. For example, a limitation of many systematic reviews is that their content and format are frequently not useful to decision makers and reasons for heterogeneity are not frequently explored leading to inconclusive and non-specific results [8-10]. While guidance exists describing what to include in reports of systematic reviews (e.g., the PRISMA statement) [11], characteristics of the intervention that are necessary to apply their findings are frequently not provided [12-14]. This has led to some preliminary work on how to extract clinically relevant information from systematic reviews [15]. Furthermore, systematic reviews commonly show substantial heterogeneity in estimated effects, possibly due to methodological, clinical or other unknown features (e.g., missing or unpublished data) in the included trials [16]. But, the reasons for or sources of heterogeneity are infrequently explored [9,10]. Thus, systematic reviewers need to consider how best to handle sources of heterogeneity [1].

While guidance exists on the assessment and investigation of methodological [1] and statistical heterogeneity [1,17], little attention has been given to clinical heterogeneity [18]. The purpose of this project was to develop consensus and empirically based recommendations for investigating clinical heterogeneity in systematic reviews.

## Methods

We used a modified Delphi method in which we contacted participants by phone, convened a face-to-face focus group, and finally asked for post-meeting feedback on the completed manuscript.

First, we compiled a list of participants with expertise or an interest in clinical heterogeneity who met one or more of the following inclusion criteria: 1) publication of guidance on how to investigate aspects of clinical heterogeneity in systematic reviews of clinical trials; 2) publication of a manual or handbook for performing systematic reviews; 3) publication of a systematic review of guidelines for performing investigations of heterogeneity in systematic reviews; 4) publication of reporting guidelines for systematic review of clinical trials; 5) membership in the PRISMA Group or one of the Cochrane Collaboration Handbook editors. These individuals were identified by reviewing published papers, reports [18] and conference abstracts. Next, we contacted individuals by e-mail to determine their interest in participating.

Those individuals who agreed to participate scheduled pre-meeting phone calls with one of the investigators (JG). To generate discussion items for the face-to-face meeting, each participant was asked, “What procedures or covariates do you deem to be required for investigating clinical heterogeneity between or within clinical trials in systematic reviews (qualitative or quantitative)?” Individuals were asked for empirical or logical reasoning for each suggestion as well as possible citations. After contacting each individual, recommendations were grouped by theme together with their rationale and operational definitions. In May and June 2011, we conducted phone calls with participants.

The face-to-face group meeting was led by one investigator (JG) during a two-day meeting. On June 3^rd^ and 4^th^, 2011, we held the face-to-face focus group meeting in Ann Arbor, in which a total of 18 participants attended and participated. They were an international group (Canada, USA, United Kingdom, Germany, and Austria) from several disciplines, including clinical research, epidemiology, statistics, methodology, surgery, clinical trials, and social science (see Additional file 1 for a list of all participants). Participants were reminded during all discussions that we were seeking recommendations specifically associated with investigating clinical heterogeneity in systematic reviews. During day one we gave a background for the project and the results of a recent methodological review in the area [18]; we also presented the results of the pre-meeting item-recommendation generation phase. We then asked for any additional new items to be added to the discussion items. Next, we proceeded to discuss each item in turn, debating the need for each and presenting empirical evidence where available. It was reiterated several times that the goal of the meeting was to generate a list of recommendations. There was an open discussion of each item during which clarifications, opinions, justifications, operational definitions and new ideas were expressed. Day two continued with this round table discussion and debate. On each day, we also included small-group break-out sessions. During day one, the purpose of the break-out session was to discuss items or topics that may not have been covered well in the discussions thus far and to make recommendations to the larger group on items that required attention. During day two, the break-out session was focused around providing a summary of the most relevant and significant recommendations arising from this meeting. Once we had discussed all items and a formal set of recommendations were agreed upon, in a preliminary form, we adjourned the meeting.

After the meeting, we drafted a manuscript describing in brief the meeting results. The manuscript draft was circulated to all meeting participants and several other individuals for their review and feedback. The manuscript was then revised incorporating all participants feedback.

We received ethics approval from the University of Michigan: HUM00043487. This study was funded by the National Library of Medicine: NIH Grant 5R21LM010832-02. The funding body had no role in the design, implementation or interpretations of the results of this project.

## Results

Below we begin with a brief discussion of the agreed-upon definition of clinical heterogeneity, elaborate on each specific recommendation and we present a table that includes a summary of the group’s 12 recommendations.

### Definition of clinical heterogeneity

Clinical heterogeneity, in the context of systematic reviews, can be defined as differences in clinically related characteristics that can give rise to variations in pooled treatment effects estimates. Using the vocabulary of this paper, clinical heterogeneity can be thought of as clinical variability that results in true effect heterogeneity. The group agreed that although the term “clinical heterogeneity” does not clearly represent the underlying concept, the term is pervasive in the literature and therefore should continue to be used. Though the term “clinical” may appear to relate specifically to a patient, it is the wider clinical context (e.g., patient, physician, treatment, etc.) that is inferred in the above definition. Examples of clinically related variables include:

•Patient/participant characteristics: age, sex, baseline severity, genetic diversity, psychosocial aspects of the population (e.g., equity, socioeconomic status, gender)

•Treatment/intervention characteristics: dose, timing, route, personnel, level of training, comparator (e.g., other treatment or no treatment)

•Outcome/measurement characteristics: type of event, outcomes measure, timing, effect estimate

•Study setting: time of year, geographic setting, where data collected

#### Recommendations for investigating clinical heterogeneity in systematic reviews

Table 1 provides an overview of the recommendations that resulted from our meetings. We present the category or topic of the recommendation and a description of what the term(s) refer to as well as relevant references.

**Table 1:** Recommendations for investigating clinical heterogeneity in systematic reviews

**Recommendation category**	**Summary description**
Review team	It is recommended to have at least one or two individuals with clinical expertise, and at least one or two individuals with methodological expertise in systematic reviews/meta-analyses and on the type of study designs you are including [19,20]. The team should recognize their own biases and attempt to compensate by including members with a wide range of (potentially conflicting) beliefs.
Planning	All investigations of clinical heterogeneity should ideally be pre-planned *a priori* and not be driven by observing the data [1,17,21-35]. But, methods for looking at data to identify unanticipated variables of interest (i.e., post-hoc investigations) need to be pre-specified, as well (e.g., looking at summary tables, graphical displays) [24,27,28,32,36]. Describe the following: which variables you will investigate, how this will be done, when you will perform these investigations, and how results will be interpreted and incorporated into your results and conclusions.
Rationale	Variables should have a clear scientific rationale for their role as a treatment effect modifier (e.g., pathophysiological, pharmacologic, evidence from prior research, clinical experience) [1,7,17,20,26,27,32-34,37],[38]. Exercise parsimony in defining variable choices [1,20,28,33,39], and consider that if variables are not reported, this may be due to an under reporting problem in primary studies. That is, not finding an effect for clinically relevant variables does not imply a consistency of effect [20].
Types of clinical variables to consider	*Patient level*: Age, baseline disease severity, sex, gender, ethnicity, comorbidities, genetic, other psychosocial variables, and other important features of the disease [2,3,7,16].
	*Intervention level:* Dose/strength/intensity of treatment, duration of treatment, brand/manufacturer, co-interventions, timing, route of administration, compliance, clinician training, implementation, other [1,2,4,5,8,12].
	*Outcome level:* Event type, outcome measure type, outcome definition, length of follow-up, timing of outcome measurement(s) [1,2,4-6].
	*Other:* Research setting, geographical issues, length of follow-up [1,3,4].
Role of statistical heterogeneity	Reviewers should think through all potentially relevant variables to explore and not rely on statistical measures of heterogeneity to justify such investigations [1,20,40,41]. Clinical heterogeneity related to *specific* individual factors could be present even in the absence of a significant statistical test for the presence of heterogeneity (e.g., Cochran’s Q test) [24,27,31,36].
Plotting and visual aids	Consider using graphical displays of data from trials to help identify potential clinical reasons for heterogeneity. Examples of plotting and visual aids of the data include: summary data sheets [27], forest plots [27,28,31,32,42], L’Abbé plots [24,32,43], funnel plots [24,44], Galbraith plots/radial plots [32], influence plots [24,45,46], dose/response curves [4], multidimensional scaling [47], and heat maps [48,49]. Reviewers should be careful to avoid data dredging while using these methods of data display.
Dealing with outliers	When there are individual trials that are clear outliers, attempt to determine why and consider a sensitivity analysis where this/these trial(s) are eliminated and observe how the effect estimate changes. One may also consider an influence analysis, in which the effect of deleting individual studies from the analysis on the overall estimate is explored.
Number of investigations to perform and variables to explore	Use parsimony as a guide to such investigations. A rule of thumb for the number of trials is that there should be close to ten trials when working with summary or aggregate patient data (APD) or ten individuals per variable, when working with pooled or individual patient data (IPD) [49-52]. Consider making a hierarchy of clinically related variables and investigate only those variables for which your rationale and power are sufficient.
The use of APD vs. IPD	*APD* = summary or aggregate data from trials only. This is subject to ecological bias [30,51,53-55]—that is, investigations of trial-level variables are valid (e.g., dose, duration), while investigations of patient-level variables are not (e.g., age, baseline severity).
	*IPD* = Original individual data on each patient. This type of data is valid for both trial-level and patient-level variables [16,22,34-36,56-60]. But, one must control for baseline difference between the patients across trials.
	Consider contacting authors and reviewing protocols of primary studies where available. Obtaining IPD for investigating clinically related patient-level variables is ideal.
The role of the best evidence syntheses	Pre-plan to use a best evidence synthesis if the studies are not reasonably combinable. Be sure to pre-plan criteria to determine combinability of included trials (e.g., sufficiently similar patient groups). This approach can also be useful for exploring differences between/within the included studies. Several recommendations for how to perform a narrative synthesis, for using levels of evidence or performing a best evidence synthesis exist in the literature e.g., [61-63].
Statistical methods	Many statistical methods are available for investigating the association of study findings with clinically related variables, including frequentist, Bayesian and mixed methods. Stratification and various forms of meta-regression can be useful. We recommend consulting respected texts and individuals with expertise in the statistical methods of meta-analyses and explorations of heterogeneity, especially meta-regression [23,27,28,32,35].
Interpretation of findings	Results are generally observational and thus hypothesis generating only [1,23,24,28,33,53]. Authors should express the validity of and confidence in their findings. When interpreting results of these investigations it is suggested to consider: confounding, other sources of bias (e.g., publication, misclassification, dilution, selection) [20,32], magnitude and direction of effect and CI [1,20], and thinking through the plausibility of causal relationships [41]. It may not be appropriate to conclude that there is consistency of effect if subgroup effects are not found [20]. Authors should use their findings to make specific recommendations about how future research could proceed or build upon these results (not just concluding that “more research is needed”).
Reporting	Consider the potential for lack of reporting of data or information relating to clinical variables in the primary studies. Consider contacting the authors for missing or additional data on important clinical variables. Reviewers must be careful to report all of their proposed and actual investigations of clinical heterogeneity. The PRISMA statement should be adhered to when reporting their reviews [11].

### Assembling the review team

When preparing for writing a protocol, one must consider the membership of the review team. This of course depends on the research question and all of its components: The population of interest, the intervention or exposure, the control group if any, the outcome of interest and the study design. In addition, when considering investigations of heterogeneity, and specifically clinical heterogeneity, the choice of review team members should be qualified by the abilities to provide hypotheses. Generally, it is recommended to have at least one or two individuals with clinical expertise, and at least one or two individuals with methodological expertise in systematic reviews/meta-analyses and on the type of study designs you are including [19,20]. Furthermore, the team should recognize their own biases and attempt to compensate by including members with a wide range of (potentially conflicting) beliefs concerning the hypotheses of interest.

### Planning investigations of clinical heterogeneity

Following the formation of an investigative team, one must plan, among other things, to investigate characteristics considered to be clinical in nature. That is, all investigations of clinical heterogeneity should ideally be pre-planned a priori and not be driven by observing the data [1,17,21-35]. Pre-planned and a-priori are used as synonymous terms here - they both mean before observation of the data.

One must acknowledge that systematic reviewers are themselves subject to bias, similar to clinical trialists. That is, it is generally not acceptable to observe the data first to drive which variables to investigate since one is potentially swayed by the data and not the hypotheses. This potential bias exists whether we are talking about primary studies (e.g., randomized controlled trials; RCTs) or systematic reviews. Of course, the data, or some of the data may be known by the scientists participating in the systematic review, as would be the case for scientists performing an RCT. That is, when performing an RCT it is expected that you know the prior research and if you are choosing to stratify, you may do so for variables and levels of those variables with good scientific rationale from prior research. The same is the case for prior knowledge and investigations of heterogeneity in a systematic review. But in the case of systematic reviews, it is expected that ALL of the data/studies are not observed or scrutinzed so as to bias the choice of variables to investigate. While some of the studies will likely be known, they are known for the same reasons studies are known before performing RCTs. Of note, it is likely that evidence from previous research may be referring to a study that is itself included in the systematic review, but this will not always be the case. For example, a large observational study may suggest a variable of interest, whereas a systematic review may have included only subsequent RCTs. Overall, investigations of clinical heterogeneity should ideally be pre-planned a priori and not be driven by observing the data.

As in RCTs, it is not uncommon for unanticipated variables to be explored for their influence of treatment effects in systematic reviews. That is, one may observe completed summary extraction tables in a systematic review and notice a trend in effect related to a clinical variable. It is reasonable to pre-plan, a-priori, the methods for looking at the included data to identify unanticipated variables of interest e.g., looking at summary tables, graphical displays etc.; [24,27,28,32,36]. Of course, such investigations are at a high risk of bias and should be interpreted with caution and only used for hypothesis generation. The results of any such investigations should be confirmed in follow-up research.

Overall, it is recommended that the review authors describe the following a priori: which variables will be investigated, how this will be done, when the investigations will be performed, and how the results of such investigations will be interpreted and incorporated into your results and conclusions.

### Rationale

All variables planned for investigation must have a sufficient scientific rationale for their role as a treatment effect modifier [1,7,17,20,26,27,32-34,37],[38]. That is, each variable chosen for investigation must have sufficient, explicit, rationale for why and how it was chose. Sources of such rationale may be pathophysiologic mechanisms, evidence from prior clinical research, or possibly from clinical experience. A brief description of the rationale should be given with relevant citations and empirical evidence where available. In such cases where variables are chose after looking at the all combined data from the included studies one should still attempt to give a rational for that variable choice beyond it simply being observed post-hoc. Of course, as mentioned above, these post-hoc variable choices can be problematic and should be treated with caution as bias is likely a factor. Furthermore, it is recommended to exercise parsimony in choosing variables [1,20,28,33,39]. That is, one should choose only a small number of variables of highest importance. The issue of power in these investigations is discussed below under “Number of Investigations to Perform and Variables to Explore”.

One must always be aware of the possible underreporting problem in primary studies included in systematic reviews e.g., [14]. The reporting of sufficient data associated with clinically important variables is often sub-par. That is, not finding an effect for clinically relevant variables does not imply a consistency of effect across variable that have a strong reason for being important [20]. We discuss reporting in more detail below.

### Types of clinical variables to consider

The type of variables to choose, of course, depends in the hypotheses that are being tested. But, we must be careful to try to make explicit all existing rationale on any variables, and attempt to find supporting data to suggest which effect modifiers may be important. In some cases no single clinical variable will be investigated or be of interest in a systematic review. This is reasonable since in some cases there is no reason to expect true effect heterogeneity due to a specific clinical variable. Of course, in many cases we expect that there are several variables that can be considered to be effect modifiers and that are clinical in nature. When defining which variables may be considered “clinical” we recently reviewed all relevant literature [18]. We referred closely to these findings when giving the following examples of variables one might consider.

For example, patient level clinical variables might include: Age, baseline disease severity, sex, gender, ethnicity, comorbidities, genetic, other psychosocial variables, and other important features of the disease [2,3,7,16]. Intervention level clinical variables include: Dose/strength/intensity of treatment, duration of treatment, brand/manufacturer, co-interventions, timing, route of administration, compliance, clinician training, and implementation [1,2,4,5,8,12]. Outcome level clinical variables include: Event type, outcome measure type, outcome definition, length of follow-up, and timing of outcome measurement(s) [1,2,4-6]. And finally, other clinical variables may include: Research setting, geographical issues, and length of follow-up [1,3,4].

### Role of statistical heterogeneity

Statistical heterogeneity in systematic reviews is generally defined as variations in the estimated effect between studies. Though a significant test for the presence of statistical heterogeneity (e.g., Cochran’s Q test) and a large degree of heterogeneity (e.g., I^2^ > 75%) might obligate a reviewer to look for covariates to explain this variability, a nonsignificant test or a small I^2^ (e.g., <25%) does not preclude the need to investigate covariate treatment effect interactions [24,27,31,36]. Even with low statistical heterogeneity, there may still be factors that influence the size of the treatment effect, especially if there is a strong argument (i.e., pathophysiologic or otherwise) that some variable likely does have such an influence. In particular, and related to the current paper, clinical heterogeneity related to specific individual factors could be present even in the absence of a significant statistical test for the presence of heterogeneity [24,27,31,36]. We suggest that reviewers should think through all potentially relevant variables to explore and not rely on statistical measures of heterogeneity to justify such investigations [1,20,40,41].

### Plotting and visual aids

When one is examining data from the included studies in a systematic review there are several plotting and visual methods that appear to be promising. We recommend that systematic reviewers consider using graphical displays of data from trials to help identify potential clinical reasons for heterogeneity. Examples of plotting and visual aids of the data include: summary data sheets [27], forest plots [27,28,31,32,42], L’Abbé plots [24,32,43], funnel plots [24,44], Galbraith plots/radial plots [32], influence plots [24,45,46], dose/response curves [4], multidimensional scaling [47], and heat maps [48,49]. The citations associated with these methods give excellent guidance on how to implement and interpret them.

There are potential drawbacks of using such methods after inspecting the data. That is, one may use a plethora of plotting and visual aids until an important effect for a clinical variable is revealed. Thus reviewers using these should be careful to avoid data dredging while using these methods of data display.

### Dealing with outliers

When there are individual trials that are clear outliers, we recommend that systematic reviewers attempt to determine why and consider a sensitivity analysis where this/these trial(s) are eliminated and observe how the pooled effect estimate changes. One may also consider an influence analysis, in which the effect of deleting individual studies from the analysis on the overall estimate is explored. Simply eliminating outliers is not a recommended practice as this then biases the study inclusion and resulting data.

### Number of investigations to perform and number of variables to explore

An often cited rule of thumb for the number of trials is that there should be close to ten trials when working with summary or aggregate patient data (APD) or ten individuals per variable, when working with pooled or individual patient data (IPD). This rule of thumb derives from work done in regression analyses in primary studies [50-52]. Additional studies have looked at this question as well [64-67]. These studies describe a required number of 4 to 20 events per variable depending on the type of regression method and data structure being used. But all of this evidence is related to primary studies and regression analyses therein - not meta-regression. To our knowledge, only 3 studies have looked at the type 1 error rate in meta-regression, but none of these tested the required number of events per variable [26,68,69]. Thus, this is a rule of thumb, a heuristic, a general guiding rule, and not supported by empirical evidence. What appears to be clear is that the smaller number of included studies, the more statistical heterogeneity, and the more variables explored, the higher the type 1 error rate [26,68,69]. Several methods are proposed to quell this [26,68,69].

We also recommend that systematic reviewers consider making a hierarchy of clinical variables of interest and investigate only those variables for which your rationale and power are sufficient. Overall we suggest one use the principle of parsimony or Ockam’s razor– loosely translated as “do not multiply entities beyond the extent necessary to explain a given phenomenon” - as a guide to any such investigations.

### The use of APD vs. IPD

It was our intent that the recommendations listed here would be relevant to both APD and IPD meta-analyses. But, we reasoned that some clarification of the power and utility of each type of data should be described.

When data are collected from all participants included in the trials that are themselves included in a systematic review or meta-analysis, we term this individual patient data (IPD). This data source has the obvious advantage of allowing for valid investigations of clinical heterogeneity for both trial-level and patient-level variables [16,22,34-36,56-60]. But, one must be careful to control for baseline differences between the patients across trials as this can bias the overall effect estimates in any heterogeneity investigations. Obtaining IPD for investigating clinically related patient-level variables is ideal.

Aggregate patient data (APD), or summary patient data from trials is by far the most common source of data included in systematic reviews and meta-analyses. One obvious problem with APD is that it is subject to ecological bias [30,51,53-55]—that is, while results of investigations of trial-level variables that do not vary across patients may be are valid (e.g., dose, duration of treatment), results of investigations of patient-level variables (e.g., age, baseline severity) or trial level variables that vary between patients (e.g., follow-up time) are subject to ecologic bias. Thus, systematic reviewers must be cognizant of the potential drawback of both IPD and APD meta-analyses.

We suggest that systematic reviewers consider contacting authors and reviewing protocols of systematic reviews, where available, to determine if plans for investigating particular clinical variables changed in any way that be deemed biased.

### The role of the best evidence syntheses

When performing a systematic review it may seem unreasonable to statistical combine the data from the included studies for a variety of reasons including a lack of or missing data and substantial heterogeneity between the studies. When there is substantial heterogeneity it can be ignored and a meta-analysis conducted with a fixed-effects or random-effects model (incorporating heterogeneity), one can attempt to explain the heterogeneity through subgroup analyses, meta-regression or other techniques, or one can perform a best-evidence synthesis. A best evidence synthesis entails looking at the study quality, effects sizes and directions across included studies to determine where possible effects are present. Generally, a set of criteria are used to guide such judgments and these qualities are discussed in the systematic review itself and conclusions are made on the overall evidence [1].

The Cochrane collaboration recently adopted the Grades of Recommendation, Assessment, Development and Evaluation (GRADE) criteria to aid in such assessments. The GRADE criteria rate the body of evidence for each outcome separately on: the types of study (randomized vs nonrandomized), risk of bias (study quality), publication bias (missing studies/small study effects), imprecision (variability), inconsistency (similarity in point estimates) and indirectness (heterogeneity) [1].

We suggest that systematic reviewers planning to perform a best evidence synthesis pre-plan their methods for doing so. That is, we recommend they pre-plan how to determine combinability of included trials (e.g., sufficiently similar patient groups) and for exploring differences between/within the included studies. Several recommendations for how to use the GRADE approach, performing a narrative synthesis, for using levels of evidence, or performing a best evidence synthesis exist in the literature e.g., [1,61-63].

### Statistical methods

Many statistical methods are available for investigating the association of study findings with clinically related variables, including frequentist, Bayesian and mixed methods. As noted in a recent publication, the number and sophistication of techniques is constantly growing [18]. Here we will briefly describe four available options—subgroup analyses, meta-regression, the analogue to the analysis of variance (ANOVA), and meta-analyses of subgroups from primary studies.

Subgroup analyses involve separating trials into groups relative to levels some characteristic (e.g., intervention duration) and performing separate meta-analyses for each group. This test provides an effect estimate within subgroups and a significance test for that estimate. As more subgroup analyses are done the likelihood of type 1 errors increase. There are some suggestions in the literature for how to control for this e.g., [18]. To test for differences between subgroups a moderator analysis must be done. Moderator analyses include meta-regression and the analogue to the ANOVA, among other techniques e.g., Z test; [22]. Meta-regression is similar to standard regression and is used to assess the influence of independent variables (e.g., intervention type) upon the dependent variable, the pooled treatment effect estimate in a meta-analysis. Many separate types of modeling strategies are available for meta-regression e.g., [70]. Next, the analogue to the ANOVA examines the difference in the effect between categorical levels of some variable using statistical methods that are identical to standard ANOVA e.g., [28].

Finally, it is acceptable combine subgroup effects from within studies using separate meta-analyses e.g., [23]. Of course, each separate meta-analysis done increases the chance of type one errors, similar to performing multiple subgroup analyses within a meta-analysis. Also, one should be aware that subgroup analyses in a primary study can still suffer from ecologic bias. Whereas subgroups that were preplanned and stratified in a primary study, for example prior to randomization in an RCT, can also be combined in meta-analyses, which have much more validity than post-hoc, post randomization, subgroup formation. Also, one must still be aware of the role of additional variables beyond that which patients were stratified on or divided into subgroups on the basis of. That is, while subgroup effects may not be found, it does not rule out effects for other variables.

We recommend that systematic reviewers attempting to perform these analyses consult textbooks and individuals with expertise in the statistical methods of meta-analyses and explorations of heterogeneity e.g., [22,23,27,28,32,35].

### Interpretation of findings

It was a consensus among the participants that the results of most investigations of clinical heterogeneity are generally observational and thus hypothesis generating only [1,23,24,28,33,53]. We recommend that the systematic reviewers should express the validity of and confidence in their findings of investigations of clinical heterogeneity. We also recommend that when interpreting the results of such investigations to consider confounding, other sources of bias e.g., publication, misclassification, dilution, selection; [20,32], magnitude and direction of effect, variability in effect [1,20], and thinking through the plausibility of causal relationships for potential influential clinical variables [41].

Furthermore, as briefly mentioned above, it may not be appropriate to conclude that there is consistency of effect when subgroup effects are not found [20]. Also, authors should use their findings to make specific recommendations about how future research could proceed or build upon these results and not simply and generally conclude that “more research is needed”. That is, when effects for clinical variable are found, or even trends in effect on pooled estimates, when plausible, systematic reviewers should recommend a confirmation of such findings in follow-up research.

### Reporting

It has been repeatedly recognized that there is poor reporting in systematic reviews and in primary studies on which they are based e.g., [11,14]. That is, in many circumstance the data or information required to perform an investigation of some clinical variable may not be completely reported across any or all studies included in a systematic review. Thus, we recommend that systematic reviewers consider the potential for lack of reporting of data or information relating to clinical variables in the primary studies included in their reviews. Because of this, one should consider contacting the authors for missing or additional data on important clinical variables.

Furthermore, systematic reviewers must be careful to report all of their proposed and actual investigations of clinical heterogeneity. Some evidence suggests that systematic reviewers are currently not doing this and that this could lead to confusion from those reading and interpreting these investigations e.g., [71]. Reporting guidelines exist for generic meta-analyses and for meta-analyses of observations studies [11,72]. The PRISMA or MOOSE statement should be adhered to when reporting systematic reviews and meta-analyses [11,72].

## Discussion

We conducted a consensus development study, in the form of a modified Delphi process, to develop recommendations for investigating clinical heterogeneity in systematic reviews of controlled studies. We expanded upon findings from the empirical literature and added several additional recommendations from a diverse group of experts (Table 1). In particular we provide a definition of clinical heterogeneity that can be used by systematic reviewers and methodologists. This definition focuses on characteristics that are clinically related and that influence treatment effect estimates. It also focuses squarely on variables as reflected in the magnitude of statistical relation between it and an outcome variable. Overall, we expect that these recommendations will aid systematic reviewers in investigating differences among and within studies and further improve the applicability of systematic review findings.

To create our recommendations, we used a consensus method, informed by empirical literature and expertise. That is, some recommendations are not supported by empirical evidence and therefore have unknown validity for investigating clinical heterogeneity in systematic reviews. We recommend that where possible these recommendations be tested and revised as new knowledge becomes available. However, we made every effort to identify evidence for these recommendations and circulated the findings to a wide audience of experts, beyond those involved in the consensus process, to comment on and revise our findings. In particular, we referred to a comprehensive methodological review of published recommendations for investigating clinical heterogeneity that was completed prior to the current project [18]. Therefore, we expect that these recommendations represent a well-grounded set of ideas to aid systematic reviewers in investigating clinical heterogeneity. Also, while we focus in the paper on the concept of clinical heterogeneity, many of these recommendations apply to investigating other sources of heterogeneity (e.g., methodological heterogeneity).

While there are many articles and resources in the literature providing recommendations for investigating clinical heterogeneity in systematic reviews, few existing resources include a relatively comprehensive set of recommendations on the topic [1,24,28]. In particular, the Cochrane Handbook, which is regularly updated, does provide some of the details we describe in Table 1[1]. Other resources have also described methods for performing statistical investigations of clinical heterogeneity e.g., [32].

## Conclusions

Our recommendations are intended to assist investigators during several stages of completing a systematic review. In particular, these recommendations will help guide the planning of investigations of clinical heterogeneity, implementing such plans, and reporting the findings. We suggest that such investigations, while often observational, may improve the applicability of the findings and their utility for decision-making.

We recommend that empirical work be carried out to test each of these recommendations. We also welcome critical feedback so that we may improve and further develop these ideas to aid systematic reviewers and end users of these studies. Finally, we hope that scientific groups, editorial boards, and funding agencies consider these recommendations when implementing, reviewing, and funding systematic reviews. These efforts will improve the validity and reliability of investigations of clinical heterogeneity.

## Competing interests

The authors declare receiving no support for this study from any organization, have no financial relationships with any organizations that might have an interest in the study, and have no other relationships or activities that could appear to be conflicts of interests.

## Authors’ contributions

JG, HM and DM conceived of the design, applied for funding, obtained ethics approval, organized the meeting, interpreted the findings and wrote and edited the manuscript. DA, JB, SC, PMcC, and XS contributed to and edited the manuscript. All members listed in Additional file 1 contributed to the content of this paper. All authors read and approved the final manuscript.

## Pre-publication history

The pre-publication history for this paper can be accessed here:

http://www.biomedcentral.com/1471-2288/13/106/prepub

## Supplementary Material

Additional file 1 Participants in the Ann Arbor Clinical Heterogeneity Consensus Group. Click here for file
