# Supplementary material for: Consensus-based recommendations for investigating clinical heterogeneity in systematic reviews
Source: BMC Med Res Methodol. 2013 Aug 30;13:106. doi: 10.1186/1471-2288-13-106 (PMC3847163; doi:10.1186/1471-2288-13-106)
Supplement: Additional file 1 — Participants in the Ann Arbor Clinical Heterogeneity Consensus Group. [file 1471-2288-13-106-S1.docx]

**Additional file 1: Participants in the Ann Arbor Clinical Heterogeneity Consensus Group**

*Doug Altman*, DSc, Center for Statistics in Medicine, University of Oxford, Oxford, UK; *Jesse Berlin*, ScD, Research and Development, Johnson and Johnson Pharmaceutical, Philidelphia, PA, USA; Joseph Beyene, MSc, PhD, Clinical Epidemiology and Biostatistics, McMaster University, Hamilton, ON, CAN; *Claire Bombardier*, MD, Institute of Health Policy Management and Evaluation, University of Toronto; Division of Rheumatology and Department of Health Policy, Management, and Evaluation, University of Toronto; Division of Clinical Decision Making & Health Care, Toronto General Research Institute, University Health Network; Institute for Work and Health; Mount Sinai Hospital, Toronto, ON, CAN; *Madeline Boscoe* RN, DU, REACH Community Health Centre, Vancouver, BC, CAN; *Stephanie Chang*, MD, MPH, Agency for Healthcare Research and Quality, Rockville, MD, USA; *Kay Dickerson*, MA, PhD, Johns Hopkins Bloomberg School of Public Health, Johns Hopkins University, Baltimore, MA, USA; *Joel J. Gagnier*, ND, MSc, PhD, Departments of Orthopaedic Surgery, Department of Epidemiology, School of Public Health, University of Michigan, Ann Arbor, MI, USA; *Gerald Gartlehner*, MD, MPH, Department for Evidence-based Medicine and Clinical Epidemiology, Danube University, Krems, AUS; *Scott Haldemann*, DC, MD, PhD, Department of Epidemiology, Department of Neurology, University of California, CA, USA; *Peter McCulloch*, MD, Centre for Evidence Based Medicine, University of Oxford, Oxford, UK; *David Moher*, MSc, PhD, Clinical Epidemiology Program, Ottawa Hospital Research Institute; Department of Epidemiology, University of Ottawa, Ottawa, ON, CAN; *Paul Montgomery*, MSc, Centre for Evidence Based Intervention, Oxford University, Oxford, UK; *Hal Morgenstern*, PhD, Departments of Epidemiology and Environmental Health Sciences, School of Public Health, University of Michigan, Ann Arbor, MI, USA; *Mark Simmonds*, MA, PhD, Centre for Reviews and Dissemination, University of York, York, UK; *Paul Shekelle*, MD, MPH, PhD, Quality Assessment and Quality Improvement Program, RAND Health; UCLA School of Medicine, Los Angeles, CA, USA; *Xin Sun*, PhD, Kaiser Permanente Center for Health Research and Oregon Evidence-based Practice Center, Oregon Health Sciences University, Portland, OR, USA; *Thomas Trikalinos*, MD, PhD, Tufts Evidence-based Practice Center, Institute for Clinical Research and Health Policy Studies, Tufts Medical Center, Boston, MA, USA.
